# Das Data Monitoring Committee – State of the Art und Perspektiven für die Zukunft

**DOI:** 10.1007/s00103-020-03212-w

**Published:** 2020-09-08

**Authors:** Thomas Fischer

**Affiliations:** Fachgebiet Klinische Prüfung/Klinik, Bundesinstitut für Arzneimittel und Medizinprodukte, Kurt-Georg-Kiesinger-Allee 3, 53175 Bonn, Deutschland

**Keywords:** Data Monitoring Committees, Statistische Analyse, BfArM-Datenbank, Innovative Studiendesigns, COVID-19, Data monitoring committees, Statistical analysis, BfArM databank, New study designs, COVID-19

## Abstract

**Hintergrund:**

Die klassische randomisierte und kontrollierte klinische Prüfung steht mit komplexen Studiendesigns und Disease-Interception-Konzepten vor neuen Herausforderungen. Dabei können Data Monitoring Committees (Datenüberwachungskomitees [DMCs]) eine zentrale Funktion übernehmen, wenn eine fachgerechte Integration in den methodischen Ablauf der klinischen Prüfungen erfolgt. Vor diesem Hintergrund haben die zuständige Bundesoberbehörde und die zuständige Ethikkommission im impliziten/expliziten Genehmigungsverfahren zu überprüfen, ob eine substanzielle Charta die Arbeitsweise des unabhängigen Gremiums abbildet.

**Ziel:**

Es wurde der Frage nachgegangen, mit welchen Häufigkeiten und Gegebenheiten DMCs in klinischen Prüfungen eingesetzt werden.

**Methode:**

Die Datenbank des Bundesinstituts für Arzneimittel und Medizinprodukte (BfArM) bildet die Grundlage für eine Datenanalyse hinsichtlich Häufigkeiten der Implementierung von Data Monitoring Committees unter verschiedenen Kriterien über einen Beobachtungszeitraum von mehr als 15 Jahren.

**Ergebnisse:**

Bei insgesamt 14.135 klinischen Prüfungen mit Arzneimitteln wurde 4152 DMCs eingesetzt. Das unabhängige Expertengremium wurde überwiegend von kommerziellen Sponsoren in der Phase III der klinischen Entwicklung integriert. Die Ethikkommissionen waren mit unterschiedlichen absoluten Häufigkeiten eingebunden.

**Diskussion:**

Die Sponsoren zeigen eine zunehmende Bereitschaft, DMCs in die methodische Durchführung von klinischen Prüfungen, insbesondere bei neuartigen Studiendesigns, zu integrieren. DMCs können eine wichtige wissenschaftliche Unterstützung sein, um die Implikationen des Coronavirus SARS-CoV‑2 auf klinische Prüfungen zu beurteilen.

## Abstract (en)

**Background:**

The classic randomized and controlled clinical trial is facing new challenges with complex study designs and disease interception concepts. For this reason, data monitoring committees (DMCs) can take on a central function if professionally integrated into the methodical procedure of clinical trials. On this basis, the responsible competent authority and the responsible ethics committee have to verify the substantial charter document in the implicit/explicit approval procedure reflecting the working process of the independent committee.

**Objectives:**

The frequencies and conditions under which DMCs are used in clinical trials was investigated.

**Methods:**

The database of the Federal Institute for Drugs and Medical Devices (BfArM) was the basis for statistical analysis concerning the frequency of implementation of data monitoring committees with different criteria over an observation period of more than 15 years.

**Results:**

In total, 4152 DMCs have been used in 14,135 clinical trials with drugs. The independent expert committee was mostly integrated by commercial sponsors in phase III of the clinical development. The ethics committees were involved with different absolute frequencies.

**Discussion:**

Sponsors demonstrate an increasing willingness to integrate DMCs in the methodical conduct of clinical trials especially in the case of new study designs. DMCs could be an important scientific aid in order to assess the implications of coronavirus SARS-CoV‑2 on clinical trials.

## Einleitung

Angesichts innovativer Ansätze in der Arzneimitteltherapie steht auch die klassische randomisierte und kontrollierte klinische Prüfung vor einer notwendigen Weiterentwicklung, zumal die neuen individualisierten Therapiekonzepte immer häufiger komplexe Studienkonzepte als Grundlage haben, welche den Entwicklungsprozess im Phasenkonstrukt der klinischen Prüfung flexibilisieren und beschleunigen sollen. Dabei können insbesondere Data Monitoring Committees (Datenüberwachungskomitees [DMCs]) zukünftig einen wesentlichen Beitrag leisten, um bei adaptiven Studiendesigns effektive Therapieoptionen rasch zu erkennen und entweder bei bewiesener Wirksamkeit oder bei ungünstigen therapeutischen Ansätzen die klinische Prüfung frühzeitig zu beenden. Eine vergleichbare zentrale Aufgabe ist für ein DMC auch bei neuartigen Disease-Interception-Konzepten möglich, wenn die Diagnose und therapeutische Intervention bei symptomfreien Menschen vorverlagert werden, welche aufgrund von prädiktiven Biomarkern ein hohes Krankheitsrisiko aufweisen.

Vor diesen neuen Herausforderungen soll mithilfe der nachfolgenden wissenschaftlichen Forschungsanalyse ein differenzierter retrospektiver und prospektiver Ausblick für die Implementierung von DMCs in klinischen Prüfungen auf der Grundlage der globalen regulatorischen Rahmenbedingungen gegeben werden. Bei einer bereits bestehenden hohen Regelungsdichte wird mit der Anwendung der neuen EU-Verordnung Nr. 536/2014 auch die Zusammenarbeit zwischen zuständiger Bundesoberbehörde und zuständiger Ethikkommission bei der Überprüfung der Funktionalität von Data Monitoring Committees noch enger werden. Dabei hat sich die Rolle der Ethikkommissionen in den letzten Jahrzehnten grundlegend verändert. Die nationalen Ethikkommissionen sind bei klinischen Prüfungen mit Arzneimitteln vollständig in die ordnungsrechtliche Regulierung des Arzneimittelrechts integriert [1]. Mit dem impliziten/expliziten Genehmigungsverfahren sind die Bundesoberbehörde und die federführende Ethikkommission zuständig für die Genehmigung bzw. Bewertung von Anträgen für klinische Prüfungen in Deutschland. Gemäß den gesetzlichen Bestimmungen hat der Sponsor die zentrale Rolle in der Durchführung einer klinischen Prüfung inne. Neben der dritten Säule des DMCs überprüfen sowohl die Bundesoberbehörde als auch die Ethikkommission bei der Antragstellung, ob auch die zweite Säule für die Durchführung einer klinischen Prüfung – das Monitoring und das Auditsystem – adäquat in den methodischen Ablauf der klinischen Prüfung integriert wurde [2]. Wenn Änderungen für den Studienablauf durch ein DMC empfohlen werden, müssen sowohl die Bundesoberbehörde als auch die zuständige Ethikkommission im Rahmen einer substanziellen Änderungsanzeige die Vorschläge medizinisch-wissenschaftlich überprüfen und autorisieren.

Die zunehmende Bedeutung von DMCs in der klinischen Prüfung zeigt sich auch in der differenzierten Ausgestaltung der globalen regulatorischen Grundlagen. Sowohl die europäische „Guideline on data monitoring committees“ [3] als auch das amerikanische Dokument „Guidance for clinical trial sponsors – establishment and operation of clinical trial data monitoring committees“ [4] – sind im Jahre 2006 in Kraft getreten. Die japanische PMDA (Pharmaceuticals and Medical Devices Agency) hat mit einer eigenständigen „Guidance data monitoring committee“ eine vergleichbare Grundlage für die Implementierung dieses unabhängigen Gremiums geschaffen.

Unter Berücksichtigung der globalen regulatorischen Grundlagen kann die nachfolgende Forschungsanalyse mit den Angaben der Sponsoren in der Datenbank des Bundesinstituts für Arzneimittel und Medizinprodukte (BfArM) über einen Beobachtungszeitraum von mehr als 15 Jahren einen wichtigen wissenschaftlichen Beitrag zur Abbildung der realen Implementierungsrate von DMCs in klinischen Prüfungen leisten. Der genannte Zeitraum beginnt mit dem Inkrafttreten der Verordnung über die Anwendung der Guten Klinischen Praxis (GCP-Verordnung) am 14.08.2004 zur Umsetzung der europäischen Direktive 2001/20/EC bzw. der Einführung des impliziten/expliziten Genehmigungsverfahrens für klinische Prüfung von Arzneimitteln in Deutschland und endet mit dem Stichdatum 30.04.2020.

Insgesamt können für diese zeitliche Periode noch einheitliche rechtliche Rahmenbedingungen zugrunde gelegt werden, bevor mit dem aktiven Inkrafttreten der neuen EU-Verordnung Nr. 536/2014 in der Europäischen Union neue Formen der kooperativen Zusammenarbeit für die Mitgliedstaaten gelten. Diese wissenschaftliche Analyse von 14.135 klinischen Prüfungen mit Arzneimitteln mit einem absoluten Anteil von 4152 in den klinischen Ablauf integrierten DMCs unterstreicht die zunehmende Bedeutung dieses unabhängigen externen Expertengremiums in der differenzierten und fortschrittlichen Arzneimittelentwicklung. Mit den realen Erfahrungswerten werden zusätzliche praktische Empfehlungen für die zukünftige Anwendung dieses unabhängigen Gremiums in klinischen Prüfungen vor dem Hintergrund der neuen Herausforderungen gegeben. Dazu werden in den nachfolgenden Abschnitten zunächst die neuen Studienkonzepte vorgestellt, bevor über eine Darstellung der globalen regulatorischen Grundlagen die Erkenntnisse aus der internen BfArM-Datenbank näher wissenschaftlich analysiert werden. Vor dem Hintergrund der gegenwärtig laufenden klinischen Prüfungen mit COVID-19-Patienten können diese unabhängigen DMCs eine wertvolle wissenschaftliche Unterstützung für die Erhebung von belastbaren klinischen Daten sein.

### Neue Studienkonzepte – Notwendigkeit von Data Monitoring Committees

In der individualisierten Medizin soll jeder Patient unter Berücksichtigung individueller Faktoren über die gesicherte Diagnose hinaus „maßgeschneidert“ behandelt werden können. Dabei kann die klinische Prüfung von Arzneimitteln bereits eine Grundlage bilden, um entsprechende Patientengruppen für eine verbesserte Wirksamkeit bzw. Sicherheit von neuen Arzneimitteltherapien zu differenzieren. Im Gegensatz zur „sprechenden“ Medizin können mittels identifizierter Biomarker differenzierte und stratifizierte Patientengruppen gebildet werden. Dazu haben wissenschaftliche Fortschritte vor allem in der Molekularbiologie und Immunologie zur Entwicklung neuer, gezielter Therapien in erster Linie bei onkologischen Erkrankungen geführt. Für eine flexible klinische Entwicklung des Bereiches der neuartigen Therapieoptionen besteht zunehmend die Erfordernis, die klassische randomisierte und kontrollierte klinische Prüfung durch ein Repertoire verschiedener neuer Studienkonzepte zu ergänzen, um den Entwicklungsprozess von neuartigen Therapien möglichst effektiv zu beschleunigen [5].

Bei diesen komplexen Studienprotokollen sind vor allem adaptive Studiendesigns, „Umbrella-“ und „Basket-Studien“ zu nennen. Für die Beurteilung dieser neuen Therapieansätze durch die zuständige Bundesoberbehörde und die federführende Ethikkommission ist eine klare Darstellung, inkl. Fallzahlschätzung und statistischen Konzepts im Prüfplan, – ggf. in einem entsprechenden Anhang – notwendig [6]. Dabei kann der entsprechende Bewertungsprozess durch die Implementierung eines unabhängigen DMCs substanziell unterstützt werden. Vor Beginn von derartigen komplexen klinischen Prüfungen sollten in einer Charta klare Verfahrensabläufe und Verantwortlichkeiten festgelegt sein. Die unabhängigen Mitglieder des DMCs stehen in unmittelbarer Berichtspflicht dem verantwortlichen Sponsor gegenüber. Nur in Ausnahmefällen erscheint eine Korrespondenz mit der Bundesoberbehörde und/oder der Ethikkommission angezeigt. Der Ethikkommission obliegt gleichfalls die medizinisch-wissenschaftliche Bewertung des Vorhabens und sie stellt zusätzlich sicher, dass auch die Prüfungsteilnehmer vollständig und in geeigneter Form über alle Aspekte eines komplexen Studiendesigns aufgeklärt werden – insbesondere das flexible Öffnen und Schließen von Studienarmen in Abhängigkeit von neuen Erkenntnissen in der klinischen Prüfung.

Neben komplexen Studiendesigns stehen auch Disease-Interception-Konzepte für einen Paradigmenwechsel in der klinischen Entwicklung von neuartigen Arzneimitteln in der Behandlung schwerer und lebensbedrohlicher Erkrankungen. Mithilfe von Biomarkern sollen frühzeitig individuelle Veränderungen im menschlichen Organismus identifiziert werden, bevor die entsprechende Krankheit mit einer klinischen Symptomatik ausbricht. Bei diesem Konzept besteht die Zielsetzung, den Erkrankungsprozess durch eine wirksame Intervention zu unterbrechen. Bei potenziellen Patienten sind häufig keine ausreichenden therapeutischen Behandlungsoptionen vorhanden, sodass Menschen mit einem hohen Risiko gezielt in ein medizinisches Monitoring aufgenommen werden, um zum richtigen Zeitpunkt eine gezielte Intervention vorzunehmen („Interception Window“). Es ist sehr gut vorstellbar, dass die verschiedenen Therapieoptionen auch hier zukünftig von unabhängigen Experten eines DMCs überwacht werden, um für die Prüfungsteilnehmer eine möglichst hohe Sicherheit in der Erhebung der klinischen Daten zu gewährleisten.

### Globale regulatorische Grundlagen für die Implementierung

Gemäß der revidierten GCP-Guideline des International Council for Harmonisation of Technical Requirements for Pharmaceuticals for Human Use (ICH; [7]) besteht die Kernaufgabe von den unabhängigen DMCs darin, in bestimmten Abständen den Fortgang einer klinischen Prüfung, die Sicherheitsdaten sowie die Hauptzielparameter für die Wirksamkeit zu bewerten und dem Sponsor Empfehlungen zu geben, ob die klinische Prüfung fortgesetzt, geändert oder abgebrochen werden sollte. Trotz klarem Aufgabenspektrum werden mehrere synonyme Begrifflichkeiten für Data Monitoring Committees benutzt (z. B. Data and Safety Monitoring Board [DSMB], Independent Data Monitoring Committee [IDMC] und Monitoring Committee [MC]).

Die drei großen Zulassungsbehörden der ICH-Region, die Europäische Arzneimittel-Agentur (EMA), die US-amerikanische Food and Drug Administration (FDA) und die japanische PMDA, haben jeweils ein Guidance-Dokument zur Etablierung von DMCs in klinischen Prüfungen verfasst und publiziert. Dazu wurden wesentliche Grundlagen für eine differenzierte regulatorische Ausgestaltung von der Studiengruppe DAMOCLES (*DA*ta *MO*nitoring *C*ommittees: *L*essons, *E*thics, *S*tatistics) und der Weltgesundheitsorganisation (WHO) erarbeitet. Auf der wissenschaftlichen Grundlage der veröffentlichten Literatur wurde ausdrücklich eine detaillierte Charta für jedes DMC vor dem Beginn der betreffenden klinischen Prüfung empfohlen [8]. Die publizierten Vorschläge bilden die Grundstruktur des zentralen Chartadokuments ab, welches die Funktionalität des unabhängigen Gremiums mit seinen Verantwortlichkeiten und Verfahrensabläufen beschreibt [9].

Bereits seit dem Jahr 1950 wurde das Konzept von DMCs bei randomisierten klinischen Prüfungen verfolgt. Eine gute Beschreibung für eine erste regulatorische Weiterentwicklung eines Policy Advisory Board (PAB) stammt aus dem Jahr 1981. Im damaligen „Coronary Drug Project“ wurden 3 von 5 Behandlungsarmen frühzeitig beendet [10]. Erst kürzlich wurde nun von der europäischen, biostatistischen Arbeitsgruppe ein Questions-and-answers-Papier über DMCs zur weiteren Kommentierung abschließend veröffentlicht [11]. Mit 7 ausgewählten Punkten soll ein differenziertes Verständnis für die Arbeitsweise von DMCs in der Europäischen Union gefördert werden. Kritisch diskutiert werden insbesondere die beiden Fragen direkte Kommunikation zwischen kompetenten regulatorischen Behörden und einem DMC bzw. die Unabhängigkeit von Mitgliedern in der frühen Phase der klinischen Entwicklung. Während zu der ersten Problematik im Kapitel 7 des FDA-Guidance-Dokuments bereits spezielle Ausnahmesituationen für einen direkten Kontakt von Behörde und DMC skizziert wurden, soll bei der notwendigen Unabhängigkeit der Mitglieder gleichzeitig die ausreichende fachliche Expertise im Anwendungsgebiet der entsprechenden klinischen Prüfung beachtet werden. Insgesamt können durch eine enge Kooperation von Sponsor und unabhängigem DMC frühzeitig Veränderungen in der Nutzen-Risiko-Bewertung bei der Durchführung von klinischen Prüfungen erfasst werden, um rasche, adäquate Entscheidungen für die Sicherheit der Prüfungsteilnehmer treffen zu können.

Mithilfe der BfArM-Datenbank wurde nun die Fragestellung untersucht, mit welchen Häufigkeiten Data Monitoring Committees unter verschiedenen Kriterien in den Ablauf von klinischen Prüfungen integriert werden.

## Methodische Grundlagen der statistischen Auswertung

Die methodische Grundlage für die statistische Analyse der DMCs bildet eine Access-Datenbank, in welche wesentliche Merkmale einer klinischen Prüfung seit dem Inkrafttreten der GCP-Verordnung am 14.08.2004 in Deutschland eingepflegt wurden. Dabei beinhaltet das Merkmal E.8.7 die Aufnahme/Nichtaufnahme eines DMCs in die betreffende klinische Prüfung. Die entsprechenden Eintragungen mit der zuständigen Ethikkommission basieren zunächst auf den Angaben der Sponsoren im Register für klinische Studien „EudraCT“ (European Union Drug Regulating Authorities Clinical Trials Database), welche von den Verwaltungsangestellten laufend in die Datenbank hochgeladen werden. Die Verifikation erfolgte über die Assessoren im Rahmen der regulären Antragsbearbeitung, sodass sich im Einzelfall nach formaler und inhaltlicher Prüfung noch notwendige Korrekturen ergaben.

Die Erfahrungswerte über die vergangenen 15 Jahre im Umgang mit der BfArM-Datenbank haben gezeigt, dass die Sponsoren gelegentlich auch nicht unabhängige Committees unter der Bezeichnung „Data Monitoring Committee“ aufführen. In der Regel wird aber in diesen Fällen bei der inhaltlichen Bewertung der jeweiligen klinischen Prüfung eine erwartete Korrektur im Sinne eines unabhängigen DMCs von den Sponsoren umgesetzt, welches auf der Grundlage des europäischen Guidance-Dokumentes in die klinische Prüfung implementiert wird. Da vergleichbare Datenbanken, z. B. „Pharm.Net Bund“, das Merkmal „Data Monitoring Committee“ nicht erfasst haben, haben die nachfolgenden wissenschaftlichen Ergebnisse eine besondere Bedeutung.

## Ergebnisse

### Data Monitoring Committees 2004 bis 2020

Insgesamt konnten 14.135 beim BfArM beantragte klinische Prüfungen berücksichtigt werden. Mehrere gezielte Abfragen in der BfArM-Datenbank zeigen unter diesen methodischen Voraussetzungen interessante Trends über die zahlenmäßige Entwicklung von DMCs in der klinischen Prüfung auf.

**Figure 1:**
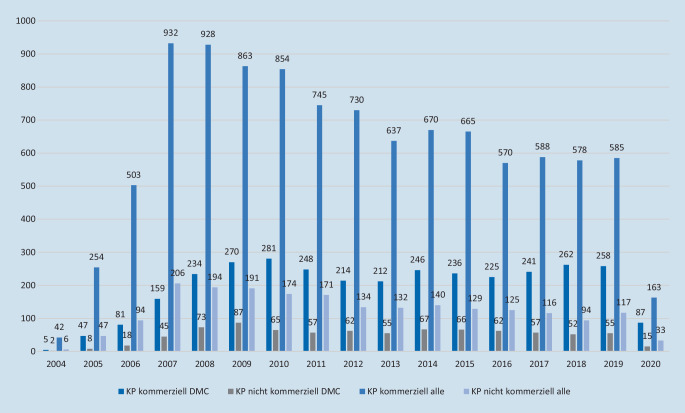

Einen Überblick zu den absoluten Häufigkeiten von DMCs sowohl in kommerziellen als auch in nicht kommerziellen klinischen Prüfungen gibt Abb. 1. Die Häufigkeit wird gemessen an der Anzahl der entsprechenden Eintragungen in der BfArM-Datenbank. Dabei kommt ein deutlicher Zuwachs in der Implementierung dieses unabhängigen Gremiums, insbesondere in den ersten Jahren, zum Ausdruck. Außerdem zeigt sich in den letzten Jahren bei einer deutlichen Abnahme der Anzahl kommerzieller klinischer Prüfungen eine entsprechende stabile Zahl dieser Prüfungen mit einem DMC. Der relativ geringere Anteil von DMCs in nicht kommerziellen klinischen Prüfungen macht auch den Aufwand deutlich, welcher mit der Einrichtung des Gremiums verknüpft ist.

In Tab. 1 spiegeln sich die regulatorischen Empfehlungen für die Implementierung des unabhängigen Gremiums in Abhängigkeit von der klinischen Phase des Entwicklungsprogramms wider. Am häufigsten werden DMCs in Phase III eingesetzt, mit der oft auch eine Zulassungsentscheidung für das Arzneimittel verknüpft ist. Aber auch in Phase II werden zunehmend DMCs aufgenommen, was möglicherweise im Zusammenhang mit beschleunigten, adaptiven Zulassungsverfahren steht.

**Table 1:** 

	Phase I	Phase I/II	Phase II	Phase II/III	Phase III	Phase IV
Anzahl DMCs in kommerziellen klinischen Prüfungen	122 (0,031)	17 (0,004)	1002 (0,256)	83 (0,021)	1810 (0,463)	90 (0,023)
Anzahl DMCs in nicht kommerziellen klinischen Prüfungen	47 (0,012)	4 (0,001)	310 (0,079)	26 (0,007)	286 (0,073)	116 (0,030)

*Anmerkung: *Es wurden nur die Zahlen mit einer eindeutigen Phasenzuordnung aus der BfArM-Datenbank aufgenommen. Die relative Häufigkeit aus der Gesamtzahl von DMCs wird bei der absoluten Zahl in der entsprechenden Klammer ausgewiesen

Aus Tab. 2 ist ersichtlich, dass DMCs in klinischen Prüfungen relativ häufig mit dem Merkmal „Multiple Member States“ vorhanden sind, d. h., dass das Gremium sicherheitsrelevante Aspekte länderübergreifend in der Europäischen Union untersucht. Da bei diesen multinationalen klinischen Prüfungen eine harmonisierte Abstimmung unter den behördlichen Institutionen und den zuständigen Ethikkommissionen erfolgt ist, kann in diesen Fällen von einem einheitlichen Verständnis der Funktionalität dieses Gremiums in Europa ausgegangen werden. Angesichts der Tatsache, dass klinische Prüfungen nur an begrenzten Probanden- bzw. Patientenkollektiven durchgeführt werden, ist dieses Merkmal von besonderem Interesse, da in der Regel bei größeren Kollektiven belastbare Entscheidungen getroffen werden können, wenn die nationalen Grenzen für die Bewertung eine untergeordnete Rolle spielen.

**Table 2:** 

	National Member State	Multiple Member States
DMCs in kommerziellen klinischen Prüfungen	230 (0,055)	3077 (0,741)
DMCs in nicht kommerziellen klinischen Prüfungen	499 (0,120)	346 (0,083)

*Anmerkung: *Die relative Häufigkeit aus der Gesamtzahl von DMCs ist in der Klammer angegeben

### Beteiligung nationaler Ethikkommissionen

**Figure 2:**
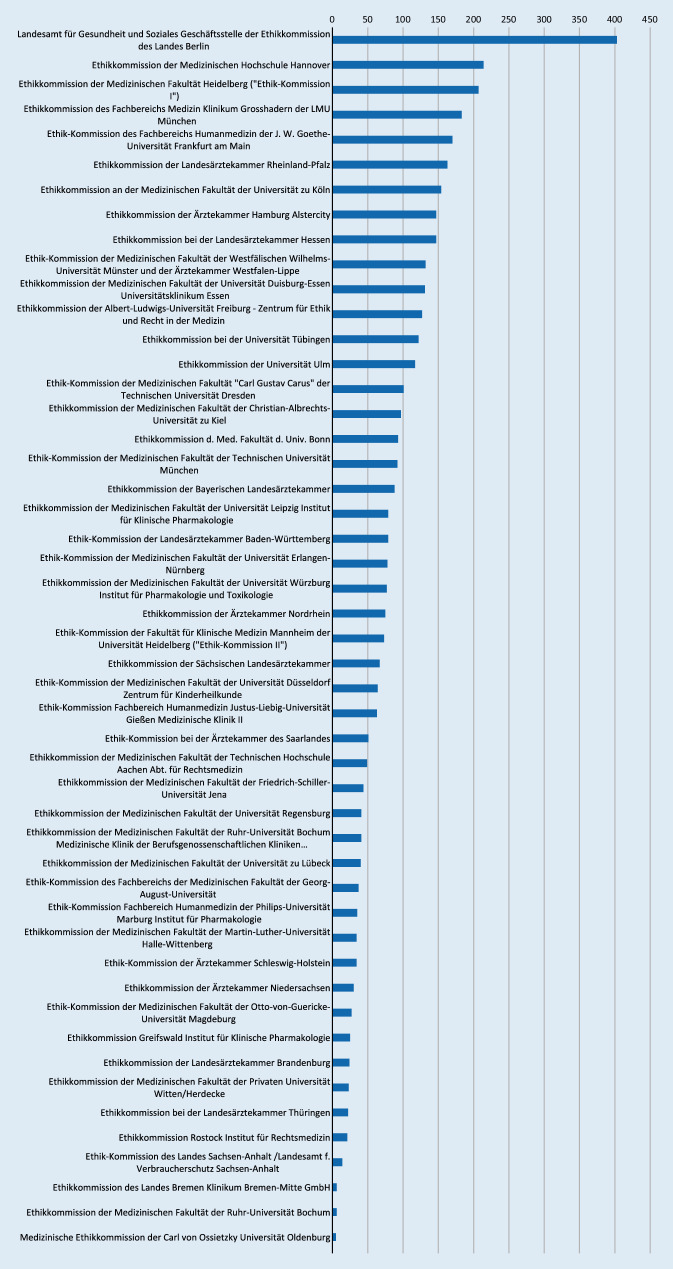

In dieser Arbeit wird auch die Beteiligung der nationalen Ethikkommissionen am Bewertungsprozess von DMCs dargestellt. Abb. 2 zeigt die entsprechenden absoluten Zahlen der jeweiligen Ethikkommissionen für das duale Genehmigungsverfahren. Die entsprechende Übersicht dient nur zur Orientierung und kann die teilweise erheblichen, strukturbedingten Unterschiede bzw. Antragszahlen bei den nationalen Ethikkommissionen nicht näher abbilden. Die bestehenden verfahrenstechnischen Unterschiede zwischen der Bundesoberbehörde und der Ethikkommission bleiben in der Darstellung gleichfalls unberücksichtigt. Dabei wurden bei den folgenden 6 Ethikkommissionen die häufigsten Eintragungen für die Implementierung eines DMCs gefunden: Landesamt für Gesundheit und Soziales Geschäftsstelle der Ethikkommission des Landes Berlin, Ethikkommission der Medizinischen Hochschule Hannover, Ethikkommission der Medizinischen Fakultät Heidelberg, Ethikkommission des Fachbereichs Medizin Klinikum Grosshadern der LMU München, Ethikkommission des Fachbereichs Humanmedizin der Universität Frankfurt und Ethikkommission der Landesärztekammer Rheinland-Pfalz.

## Diskussion

Seitdem die europäische „Guideline on data monitoring committees“ vor 14 Jahren in Kraft getreten ist, wurden diese unabhängigen externen Gremien zunehmend in den methodischen Ablauf von klinischen Prüfungen integriert. Die Data Monitoring Committees üben mittlerweile eine zentrale Funktion in der globalen klinischen Arzneimittelentwicklung aus, sodass in der ICH-Region eine differenzierte regulatorische Grundlage für alle Länder besteht [12, 13]. Mit der Einführung des impliziten/expliziten Genehmigungsverfahrens in Deutschland haben sowohl die zuständige Bundesoberbehörde als auch die zuständige Ethikkommission die fachgerechte Implementierung bei der Bewertung der Antragsunterlagen zu überprüfen.

Mit der vorliegenden wissenschaftlichen Analyse in der BfArM-Datenbank sollte der Fragestellung nachgegangen werden, mit welchen Häufigkeiten und Gegebenheiten DMCs in der klinischen Prüfung über einen Beobachtungszeitraum von über 15 Jahren in Deutschland eingesetzt wurden. Im Rahmen der statistischen Auswertung der Angaben der Sponsoren sollten auch erkennbare Trends wiedergegeben werden. Da sich die klassische randomisierte und kontrollierte klinische Prüfung mit den neuen komplexen Studiendesigns und Disease-Interception-Konzepten weiterentwickeln wird, gewinnt die Abbildung der aktuellen realen Zahlen zusätzlich an Bedeutung. Bei diesen neuen Herausforderungen in der klinischen Entwicklung von neuartigen Arzneimitteln kann vermutlich ein DMC behilflich sein, bei ausreichender Sicherheit für die Prüfungsteilnehmer frühzeitig effektive Therapieoptionen zu erkennen und die notwendigen Maßnahmen in der betreffenden klinischen Prüfung in die Wege zu leiten [14].

Zur Wahrnehmung dieser besonderen Verantwortung ist die methodische Integration eines DMCs in die klinische Prüfung stets lege artis auf der gültigen regulatorischen Grundlage und der entsprechenden Fachliteratur vorzunehmen. Eine substanzielle Charta, in welcher alle Verfahrensabläufe und Verantwortlichkeiten detailliert abgebildet sind, ist für eine Genehmigung durch die Bundesoberbehörde und die Ethikkommission mittlerweile unverzichtbar. Außerdem ist für eine qualifizierte externe klinische Überwachung der klinischen Prüfung die Unabhängigkeit der Mitglieder dieses Gremiums eine „Conditio sine qua non“ [15, 16].

Bei der Implementierung von DMCs in die Verlaufskontrolle von klinischen Prüfungen müssen sowohl klinische als auch biometrische Gesichtspunkte differenziert beachtet werden. Der verantwortliche Statistiker hat alle notwendigen Schutzvorkehrungen zu treffen, welche aus dem Eingriff in die Daten der klinischen Prüfung resultieren, um nicht die Aussagekraft der Studienergebnisse zu gefährden. Während zahlreiche statistische Arbeiten sich mit der Weiterentwicklung dieses unabhängigen Gremiums auseinandergesetzt haben [17–19], sind neue klinische Übersichtsarbeiten nur begrenzt veröffentlicht [20, 21]. Dabei sind für eine methodisch belastbare Konstruktion präspezifizierte Abbruchkriterien und ggf. Interimanalysen in den Prüfplan aufzunehmen [22, 23]. Wenn auch praktische Empfehlungen ausgesprochen werden, so fehlt doch eine umfangreiche Analyse der bisherigen regulatorischen Antrags- und Bewertungsverfahren, bei denen ein DMC eine zentrale Aufgabe übernommen hat.

Die Ergebnisse der statistischen Aufarbeitung von 14.135 klinischen Prüfungen in der BfArM-Datenbank zeigen deutlich, dass die Bedeutung von Data Monitoring Committees in der klinischen Prüfung gewachsen ist. Allein in Deutschland wurden insgesamt über einen Zeitraum von mehr als 15 Jahren 4152 DMCs im Rahmen von klinischen Prüfungen eingesetzt. Gemäß den Vorgaben der europäischen Guideline wurde dieses unabhängige Expertengremium überwiegend in der Phase III der klinischen Prüfung integriert. Die medizinischen Ethikkommissionen in der Bundesrepublik Deutschland waren in den Bewertungsprozess von DMCs eingebunden, aber mit sehr unterschiedlichen absoluten Häufigkeiten in Abhängigkeit von den jeweiligen Antragszahlen. Die relative Zunahme der Anzahl von klinischen Prüfungen mit einem DMC in den letzten Jahren ist möglicherweise Ausdruck einer höheren Bereitschaft der Sponsoren, sicherheitsrelevante Fragestellungen zusätzlich einer unabhängigen Überwachung zu unterziehen, und spiegelt auch die häufigere Notwendigkeit einer Implementierung bei der zunehmenden Komplexität von klinischen Prüfungen wider. Für die nicht kommerziellen Sponsoren wird angesichts des Aufwandes, welcher mit der fachgerechten Implementierung von DMCs verbunden ist, die Inanspruchnahme von unabhängigen, professionellen Dienstleistern innerhalb der Einrichtung angeregt [24].

## Fazit

Insgesamt bilden die statistischen Daten aus der BfArM-Datenbank die regulatorischen Erfordernisse auf Grundlage der derzeit noch gültigen Direktive 2001/20/EC ab. Mit der Aktivierung der EU-Verordnung Nr. 536/2014 wird in Zukunft die kooperative Zusammenarbeit der EU-Mitgliedsstaaten intensiver werden und vermutlich eine Weiterentwicklung in der harmonisierten Bewertung bei der Einrichtung von unabhängigen DMCs erbringen [25, 26]. Die aktuelle gravierende Coronakrise hat die Zulassung von Arzneimitteln und Impfstoffen in den Mittelpunkt der regulatorischen medizinischen Aktivitäten gerückt. Die zahlreichen Beeinträchtigungen im Rahmen der COVID-19-Pandemie betreffen sowohl laufende als auch geplante klinische Prüfungen mit Arzneimitteln. Für eine möglichst harmonisierte Vorgehensweise in der europäischen Union hat die Kommission das Dokument „Guidance on the management of clinical trials during the COVID-19 (Coronavirus) pandemic“ veröffentlicht [27]. Da insbesondere auch methodische Aspekte in der Durchführung von klinischen Prüfungen betroffen sind, hat die europäische biostatistische Arbeitsgruppe ein „Points-to-consider-Papier“ bereitgestellt, welches explizit Aufgaben und Bedeutung von Data Monitoring Committees in dieser schwierigen Krisensituation in den Mittelpunkt der methodischen Betrachtungen stellt [28]. In den laufenden klinischen Prüfungen mit COVID-19-Patienten können diese unabhängigen DMCs eine wertvolle wissenschaftliche Unterstützung sein, wenn die Implikationen des Coronavirus SARS-CoV‑2 auf die methodische Durchführung von klinischen Prüfungen mit Arzneimitteln zu bewerten sind. Aus diesem Grund wird auch die Implementierungsrate von DMCs in klinischen Prüfungen während der gegenwärtigen Pandemie zunehmen. Zukünftig werden in erster Linie komplexe Studiendesigns und Disease-Interception-Konzepte ein unabhängiges Data Monitoring Committee benötigen.
